# Transcriptomic Analysis of *Aggregatibacter* *actinomycetemcomitans* Core and Accessory Genes in Different Growth Conditions

**DOI:** 10.3390/pathogens8040282

**Published:** 2019-12-03

**Authors:** Natalia O. Tjokro, Weerayuth Kittichotirat, Annamari Torittu, Riikka Ihalin, Roger E. Bumgarner, Casey Chen

**Affiliations:** 1 Division of Periodontology, Diagnostic Sciences, and Dental Hygiene, Herman Ostrow School of Dentistry, University of Southern California, 925 West 34th Street, Los Angeles, CA 90089, USA; tjokro@usc.edu; 2 Systems Biology and Bioinformatics Research Group, Pilot Plant Development and Training Institute, King Mongkut’s University of Technology Thonburi, Bangkok 10150, Thailand; weerayuth.kit@kmutt.ac.th; 3 Department of Biochemistry, University of Turku, FI-20014 Turku, Finland; atorittu@abo.fi (A.T.); riikka.ihalin@utu.fi (R.I.); 4 Department of Microbiology, University of Washington, Seattle, WA 98109, USA; rogerb@uw.edu

**Keywords:** *A. actinomycetemcomitans*, RNA-Seq, genomic islands, core genes, accessory genes, stress, nutrient limitation, differentially expressed genes

## Abstract

*Aggregatibacter actinomycetemcomitans* genome can be divided into an accessory gene pool (found in some but not all strains) and a core gene pool (found in all strains). The functions of the accessory genes (genomic islands and non-island accessory genes) are largely unknown. We hypothesize that accessory genes confer critical functions for *A. actinomycetemcomitans* in vivo. This study examined the expression patterns of accessory and core genes of *A. actinomycetemcomitans* in distinct growth conditions. We found similar expression patterns of island and non-island accessory genes, which were generally lower than the core genes in all growth conditions. The median expression levels of genomic islands were 29%–37% of the core genes in enriched medium but elevated to as high as 63% of the core genes in nutrient-limited media. Several putative virulence genes, including the cytolethal distending toxin operon, were found to be activated in nutrient-limited conditions. In conclusion, genomic islands and non-island accessory genes exhibited distinct patterns of expression from the core genes and may play a role in the survival of *A. actinomycetemcomitans* in nutrient-limited environments.

## 1. Introduction

Gram-negative facultative *A. actinomycetemcomitans* is an oral commensal bacterium and a major causative agent of periodontitis, as well as an occasional cause of extra-oral infections [1,2,3,4]. The species comprises genetically heterogeneous strains that display differential association with periodontal health, disease, or disease progression, suggesting a pattern of strain-dependent virulence potentials including an example of a well-characterized highly leukotoxic JP2 type [5,6,7,8,9]. Beyond disease-association, genetically distinct *A. actinomycetemcomitans* strains are expected to be phenotypically distinct, which has been observed but not fully investigated [7,8,10,11]. In the landmark study by Socransky et al. [12], the correlation analysis of subgingival bacterial species identified discrete bacterial complexes (each composed of different species), suggesting either niche-sharing or metabolic interdependence of the bacteria within the complexes. Interestingly, *A. actinomycetemcomitans* serotype a and serotype b strains have different patterns of microbial associations, which may be indicative of differences in phenotypes and preferred niches.

Comparative genomics by whole genome sequencing has identified five evolutionarily distinct clades among human strains of *A. actinomycetemcomitans*, which are separated from an *A. actinomycetemcomitans* strain isolated from a Rhesus monkey and strains of a closely related oral species *Aggregatibacter aphrophilus* [13,14]. The evolutionary divergence of *A. actinomycetemcomitans* is marked by the acquisitions of strain- and clade-specific accessory genes via horizontal gene transfer. Within *A. actinomycetemcomitans*, strains of different clades may differ by as much as 20% in their genomic content. Large scale genomic rearrangements have also been noted among *A. actinomycetemcomitans* strains of different clades [15]. 

Accessory genes, including those organized into genomic islands, accounted for 14.1%–23.2% of the *A. actinomycetemcomitans* genomes. A total of 387 genomic islands of 5 Kb or larger have been identified among 31 *A. actinomycetemcomitans* strains. With a few exceptions, such as the genomic island that carries genetic determinants for cytolethal distending toxins, the functions of these islands are largely unknown. As a first step to probe the functions of genomic islands and other non-island accessory genes, this study examined the patterns of gene expression of *A. actinomycetemcomitans* in different growth conditions. We compared the gene expression profiles of a wild type biofilm-forming *A. actinomycetemcomitans* D7S-1 in enriched trypticase soy broth with yeast extract (modified Trypticase Soy Broth, mTSB) and two nutrient-limited media (RPMI and keratinocyte medium) commonly used in co-cultures of bacteria and mammalian cells. We also examined the gene expression profiles of *A. actinomycetemcomitans* D7S-1 and its isogenic non-fimbriated mutant strain D7SS in mTSB. The results showed that genomic islands and non-island accessory genes exhibited similar patterns of gene expression, and exhibited lower levels of expression than core genes in all growth conditions. However, accessory genes, including genomic islands, were highly active in nutrient-limited media. A few virulence genes, such as cytolethal distending toxin operon, were upregulated in response to nutrient-limitation. The results suggest that accessory genes, including genomic islands, may be critical for bacterial adaptation under nutrient-limitation stress.

## 2. Results

### 2.1. Core Genes, Accessory Genes, and Genomic Islands

The 2041 protein-coding genes identified in the genome of *A. actinomycetemcomitans* strain D7S-1 were first categorized into core (N = 1608) and accessory (N = 433) genes. The latter included 191 non-island accessory genes, and 242 island genes organized into 26 genomic islands of 5 Kb or larger (see Supplementary Table S1 for details of the genomic islands). Fourteen islands were found in at least one other *A. actinomycetemcomitans* strain, while the remaining 12 islands were unique to strain D7S-1 (details of the distribution of genomic islands among all sequenced *A. actinomycetemcomitans* strains will be published elsewhere). Cumulatively these 26 islands had a footprint of 275,927 bps or 12% of the D7S-1 genome. 

### 2.2. Differences in the Patterns of Gene Expression among Core, Accessory, and Island Genes

For each gene, the transcript level was the average of 3 biological replicates in each growth condition. To validate the expression levels obtained by RNA-Seq, quantitative real-time PCR (qRT-PCR) was also performed on selected genes (D7S_02294 *cdtA*, D7S_02295 *cdtB*, D7S_00244 *metF*, D7S_00604 *ltxA*). The correlations between the results obtained by qRT-PCR and RNA-Seq were shown in Supplementary Figure S1. Three of the four genes demonstrated excellent correlations between results obtained by qRT-PCR and RNA-Seq (R = 0.81–0.91). The expression levels of *ltxA* did not show good correlations between the two quantification methods probably due to experimental variables that were difficult to replicate between experiments, and this phenomenon had been observed previously [16].

The phenotypes of *A. actinomycetemcomitans* biofilms were first examined by assessing the optical density of the biofilm cultures. *A. actinomycetemcomitans* D7S-1 grew in the enriched mTSB, but did not show evidence of growth in the nutrient-limited media. We then determined the viability of *A. actinomycetemcomitans* D7S-1 by enumerating the CFU of the bacteria in the biofilms over time. The results showed that *A. actinomycetemcomitans* D7S-1 grew in the enriched mTSB (as expected), maintained its viability in RPMI without apparent growth, and showed a reduced viability in keratinocyte medium (see Supplementary Materials Figure S2 for details). Therefore, RPMI and keratinocyte medium exerted two distinct types of nutrient-limitation stress to *A. actinomycetemcomitans*.

**Figure 1:**
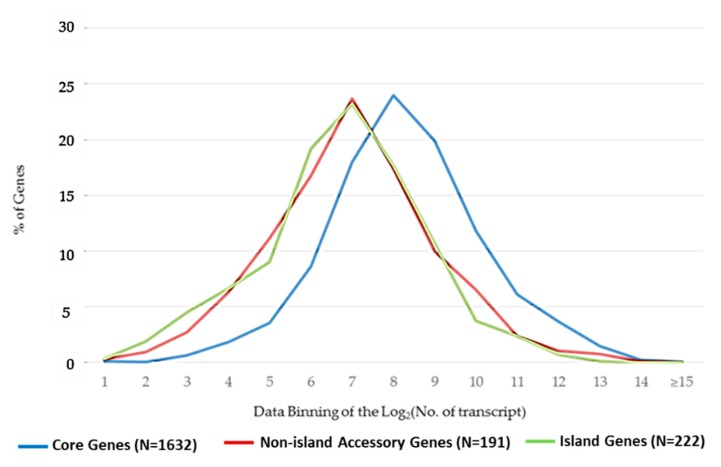
Distribution patterns of gene expression among core, non-island accessory and island genes. The expression signals were log_2_-transformed and binned 1 to ≥15 on the x-axis. 1 represents up to 2 copies of the transcript, and 2 represents up to 4 copies of transcripts et cetera.

The combined expression levels of 4 experimental conditions for core, non-island accessory genes, and island genes are shown in Figure 1. The expression levels were statistically significantly different between core and non-island accessory genes or island genes in each of the tested conditions or in all conditions (the range of the p-values was 10^−8^ to 10^−148^ by Student’s *t*-test. See Supplementary Table S2 for detailed information). The mode of the expression of non-island accessory or island genes was 2^7^ transcripts, while the mode of expression of the core genes was 2^8^ transcripts. The patterns of gene expressions were similar between non-island accessory and island genes (Supplementary Table S2). Henceforth, as appropriate, some results focused on the comparison between core and island genes.

**Figure 2:**
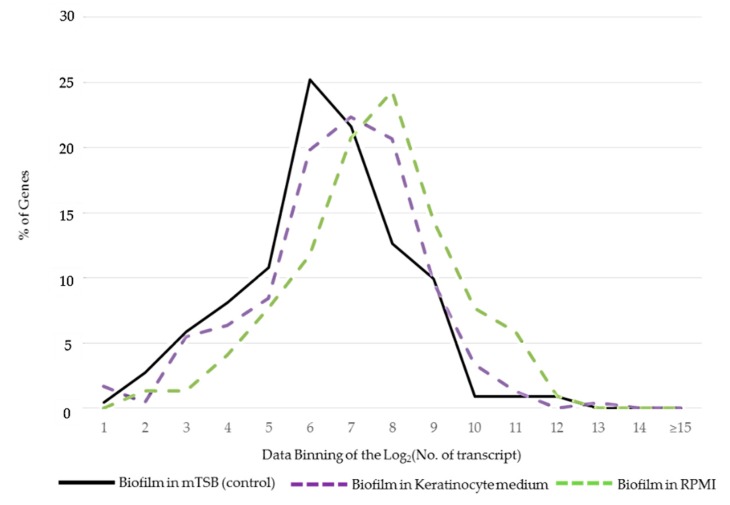
Differential expression of island genes in distinct growth conditions. The expression signals were log_2_-transformed and binned 1 to ≥15 on the x-axis.

The differences in the expression levels of core and island genes were examined in each of the four growth conditions. The mean and median expression levels of core and island genes were listed in Supplementary Table S3. We noted that island genes were expressed at higher levels in nutrient-limited media than in the enriched mTSB. Figure 2 showed that the activation of island genes was particularly pronounced when bacteria were cultured in RPMI. The results suggested that island genes were activated by stress associated with nutrient deprivation. Individual genes up- or down-regulated in these two nutrient-limited media were distinct (see Section 2.3 below).

### 2.3. Differentially Expressed Genes of A. actinomycetemcomitans

The numbers of differentially expressed genes in different growth conditions were listed in Table 1. Annotations of these genes were provided in Supplementary Table S4.

As expected, the greatest differences in gene expression levels between planktonic cells and biofilms were found in the fimbrial gene operon, which was down-regulated in planktonic cells (Supplementary Table S4). Other notable differentially expressed genes between planktonic and biofilms of *A. actinomycetemcomitans* included the genes PTS mannose transporter subunit IIAB (D7S_01753) (down-regulated), thiamine ABC transporter substrate-binding protein (D7S_02132) (upregulated), superoxide dismutase (D7S_01907) (upregulated), and peptide methionine sulfoxide reductase (D7S_00462) (upregulated). Relatively high numbers of differentially expressed genes were detected when *A. actinomycetemcomitans* strain D7S-1 was cultured in keratinocyte medium and more so in RPMI. More than 70% of island genes (as well as non-island accessory genes) of *A. actinomycetemcomitans* strain D7S-1 were differentially expressed in response to RPMI, with more than three-quarter of the genes upregulated.

**Figure 3:**
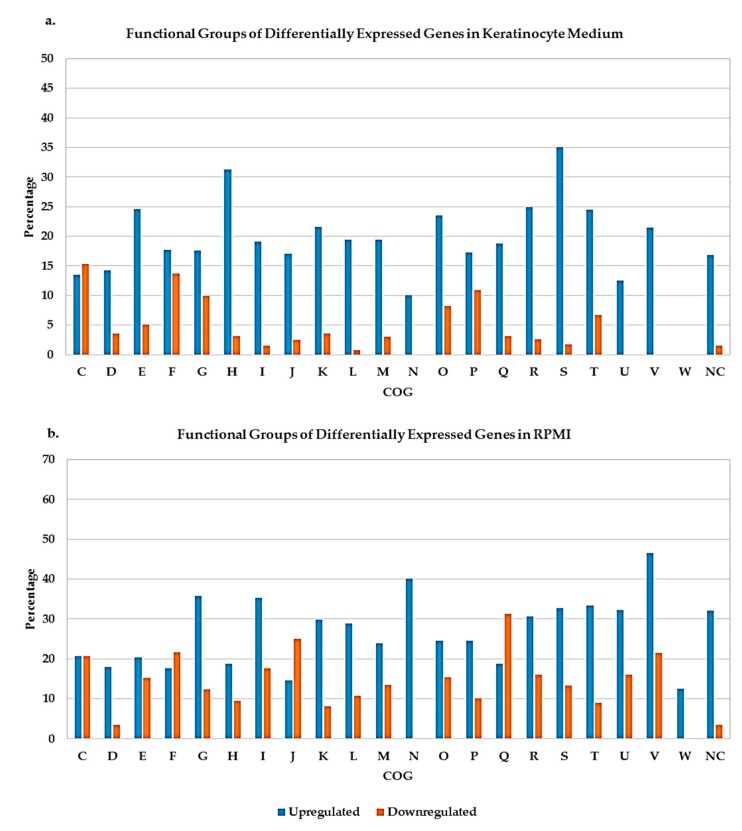
Cluster of Orthologous Groups (COG) functional categories of differentially expressed genes in keratinocyte medium (**a**) and RPMI (**b**). The y-axis is the percentage of differentially expressed genes. (**C**) Energy production and conversion, (**D**) cell cycle control, cell division, chromosome partitioning, (**E**) amino acid transport and metabolism, (**F**) nucleotide transport and metabolism, (**G**) carbohydrate transport and metabolism, (**H**) coenzyme transport and metabolism, (**I**) lipid transport and metabolism, (**J**) translation, ribosomal structure and biogenesis, (**K**) transcription, (**L**) replication, recombination and repair, (**M**) cell wall/membrane/envelope biogenesis, (**N**) cell motility, (**O**) posttranslational modification, protein turnover, chaperones, (**P**) inorganic ion transport and metabolism, (**Q**) secondary metabolites biosynthesis, transport and catabolism, (**R**) general function prediction only, (**S**) function unknown, (**T**) signal transduction mechanisms, (U) intracellular trafficking, secretion, and vesicular transport, (V) defense mechanism, (**W**) extracellular structure, (NC) not categorized.

Figure 3 showed differentially expressed genes (both accessory and core genes) in keratinocytes medium and RPMI grouped according to the Cluster of Orthologous Group (COG) categories. There were more upregulated genes compared to those that were downregulated in both media. Keratinocytes medium had about 35% of the upregulated genes belong to COG S whose functions were still unknown. About 46% of the genes in COG V (defense mechanism) were upregulated, and about 31% of genes in COG Q (secondary metabolites biosynthesis, transport, and catabolism) were downregulated genes in RPMI. These might make good targets to determine genes and pathways involved in cellular responses to the stress of nutrient limitation. 

KEGG-style metabolic networks were used to analyze biological pathways that might be affected by these differentially expressed genes. In keratinocyte medium, pathways affected included those involved in ribosomal biosynthesis, carbon metabolism, quorum sensing, microbial metabolism in diverse environments, and aminoacyl-tRNA biosynthesis. On the other hand, only ribosomal biosynthesis pathway was found to be affected significantly by the differentially expressed genes in RPMI medium. These affected biological pathways seemed to have functions that might contribute to increasing the survival likelihood of *A. actinomycetemcomitans* under the stress of nutrient limitation. 

**Figure 4:**
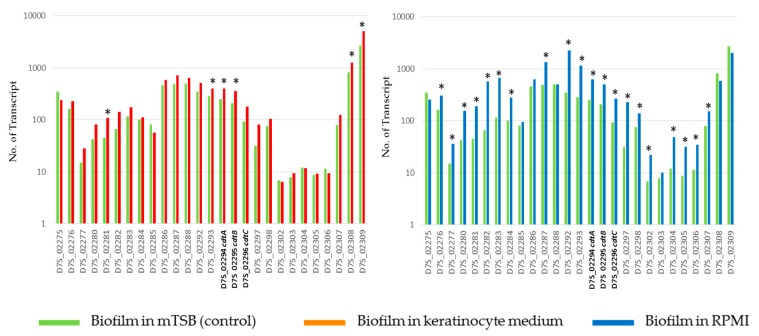
Expression levels of the 24 Kb cdt-island of D7S-1 biofilm in keratinocyte medium (**left** panel) or RPMI (**right** panel) in comparison to control in mTSB. The cdtABC are marked with bold font. See Supplementary Table S3 for annotation of other island genes. * Statistically significant at *p* < 0.05 by Student’s t-test between biofilm in keratinocyte medium and control.

Most of the genomic islands and a few selected *A. actinomycetemcomitans* virulence genes were upregulated in nutrient-limited media. These included the lipopolysaccharides biosynthesis genes in RPMI (2.3-fold) and keratinocyte medium (2.2-fold), the metal-binding heat shock protein upregulated in keratinocyte medium (1.5-fold) and RPMI (4-fold). Notably, a 24 Kb genomic island (here designated as *cdt*-island) that carried *cdtABC* was highly active and upregulated in *A. actinomycetemcomitans* exposed to RPMI, and also in keratinocyte medium to a lesser extent (Figure 4). The *cdtABC* was upregulated in both RPMI (2.5- to 3-fold) and keratinocyte medium (1.6- to 2-fold). Twenty of the 27 *cdt*-island genes, including *cdtABC* were upregulated in response to RPMI.

## 3. Discussion

Bacterial species are constantly evolving, and a prime example of this is the increasing occurrence of superbugs that are resistant to multiple antibiotics. Genomic islands are speculated to have contributed significantly to this phenomenon as they are involved in the dissemination of accessory genes, including antibiotic resistance and virulence genes. Genomic islands and the horizontal gene transfer process has been hypothesized to be a major force driving genome evolution [17,18,19,20,21]. Bioinformatics studies have also shown that genomic islands tend to carry genes that are considered novel, those with no orthologues in other species [22]. This suggests that genomic islands have been selected for adaptive and auxiliary functions [23]. 

The phenotypic variations observed in *A. actinomycetemcomitans* [5,6,7,8,9] are probably best explained by strain-to-strain variations in genome content. Core genes of *A. actinomycetemcomitans* presumably played significant housekeeping functions for the basic survival of the bacteria. In this study, the higher levels of expression of core genes and their relatively stable levels of expression in different conditions might, therefore, correlate with core genes’ functions for basic functions of *A. actinomycetemcomitans*. In this study, the core genes were identified by comparative genomics and may or may not be the same as essential genes that require experimental confirmation. A previous study utilizing rapid transposon mutant sequencing (Tn-Seq) had established the presence of essential genomes in two divergent *A. actinomycetemcomitans* strains, VT1169 and 624 strains [24]. Notably, 307 of the 319 essential genes matched to our core gene pool. Other core genes of strain D7S-1 may also be essential for growth conditions not tested in the previous study. 

The activation of *cdtABC* observed in this study may be a survival mechanism for *A. actinomycetemcomitans* in response to limited nutrients. The cytolethal distending toxin produced by *A. actinomycetemcomitans* is a trimeric holotoxin. *cdt*B is the toxin, while *cdt*A and *cdt*C facilitate the binding and entry of the toxin into the cells. The *cdt*B toxin enters the cells and traffics to the nucleus, where its DNase and lipid phosphatase activities lead to DNA damage and induce apoptosis and subsequently cell death in a variety of cell types [25,26,27,28,29,30,31]. *cdt*B may also elevate the expression level of receptor activator of nuclear factor kappa-B ligand with potential for osteoclastogenesis and bone loss [32,33]. The tissue damages and inflammatory responses triggered by *cdtABC* toxin could be a mechanism for nutrient acquisition by *A. actinomycetemcomitans*. 

There is a paucity of information regarding the regulation of *cdtABC* in *A. actinomycetemcomitans.* Shenker et al. showed evidence for the expression of a 5-gene operon comprised of *orf1*, *orf2*, *cdtA, cdtB,* and *cdtC* [25]. The functions of the upstream *orf1, orf2* (homologous to D7S_02292 and D7S_02293 on the *cdt*-island in this study) were unknown. The environmental signals that activated the *cdtABC* operon remained to be determined. Here we showed evidence that, in addition to *cdtABC* and the homologs of *orf1* and *orf2*, several other genes on the D7S-1 *cdt*-island were similarly upregulated in response to RPMI. It is unclear whether D7S-1 *cdt*-island is regulated by a single promoter to generate a long polycistronic transcript for more than five genes defined by Shenker et al. [25]. We have noted the structural dis-similarities in the *cdtABC* loci among genetically distinct *A. actinomycetemcomitans* strains. There are at least three distinct *cdtABC* variants [13]. The first was represented by the 24 Kb *cdt*-island of strain D7S-1 in this study. The second is 5 Kb in size and found in serotype b and c strains [13]. The third is represented by a 22 Kb genomic island designated as GIY4-1 described by Doungudomdacha et al. [34]. It is likely that there are multiple regulation mechanisms for *cdtABC* in genetically distinct *A. actinomycetemcomitans* strains. More details of the structure and gene compositions of the distinct *cdtABC* loci among *A. actinomycetemcomitans* strains will be published elsewhere. Moreover, *A. actinomycetemcomitans* leukotoxin expression has been shown to be regulated by growth conditions such as iron availability and anaerobiosis [35,36]. The specific environmental signals that regulate the expression of *cdtABC* in D7S-1 remain to be elucidated. 

Kawamoto et al. [37] examined the toxic activity of forty-one strains of *A. actinomycetemcomitans* on Chinese hamster ovary cells. The results demonstrated differences in cytotoxicity among strains. Serotype b and c strains appeared to be more cytotoxic than serotype a strains. Our study is limited to a single strain of serotype a and therefore the results are not comparable. It will be interesting to examine whether the differences in cytotoxicity attributed to cytolethal distending toxin could be explained by different genetic regulatory elements of *cdtABC* among *A. actinomycetemcomitans* strains. 

Several genes involved in LPS biosynthesis were found to be upregulated in poor nutrient RPMI and keratinocyte media in this study. The transcription of LPS genes is upregulated under limited nutrient availability [38]. Changing the bacterial membrane structures and their fluidity have been proposed to be a stress response that allows bacteria to limit exchanges, save energy, and survive, which may also promote biofilm formation [39,40,41,42,43]. Therefore, the observed changes in LPS gene expression may be a stress response of A. actinomycetemcomitans to starvation. Whether nutrient-limitation of A. actinomycetemcomitans leads to greater amounts of biofilm formation requires further studies. 

Differential gene expressions between *A. actinomycetemcomitans* in its planktonic and biofilm states had been observed previously [44], and our study confirmed their observations on several genes. The gene PTS mannose transporter subunit IIAB (D7S_01753) was found to be upregulated by both studies. On the other hand, the genes thiamine ABC transporter substrate-binding protein (D7S_02132), superoxide dismutase (D7S_01907), and peptide methionine sulfoxide reductase (D7S_00462) were found to be downregulated by both studies. The results may suggest different metabolic characteristics between planktonic and sessile *A. actinomycetemcomitans.*

During infection, bacteria cells are constantly exposed to various stresses of the environment, including drastic changes in temperature, pH, osmolarity, and nutritional availability. In order to survive, bacteria must cope with these stresses by regulating various gene expressions, and they are equipped with multiple mechanisms of stress responses. In the oral cavity, *A. actinomycetemcomitans* is subjected to the stress of different pH, heat, and nutrient availability [45]. In response to these environmental challenges, *A. actinomycetemcomitans* induces the expression of heat shock proteins (HSPs) that offered protection to them [46,47]. Our data provide additional evidence of HSPs involvement in *A. actinomycetemcomitans* stress response. In both keratinocyte and RPMI media, the transcription of metal-binding heat shock protein (D7S_01459) was found to be significantly upregulated. Although the importance of HSPs are evident, neither the mechanisms of protection by HSPs nor the cellular responses to the stress of nutrient limitation in *A. actinomycetemcomitans* is fully understood. Given the fact that a high percentage of upregulated genes in both RPMI and keratinocytes are those whose functions are still unknown, these genes can be targeted to study genes and pathways involved in cellular starvation responses. 

In conclusion, our study showed that patterns and levels of expression of accessory genes (island and non-island genes) are different from core genes in *A. actinomycetemcomitans*. Notably, the accessory genes were activated in nutrient-limited growth conditions. We hypothesize that accessory genes, including genomic islands, are essential for the survival of *A. actinomycetemcomitans* in the in vivo-like conditions. 

## 4. Materials and Methods 

### 4.1. Bacterial Strains and Growth Conditions

*A. actinomycetemcomitans* strain D7S-1 and its isogenic nonfimbriated mutant D7SS were routinely grown in modified Trypticase Soy Broth (mTSB) containing 3% trypticase soy broth and 0.6% yeast extract, or on mTSB agar (mTSB with 1.5% agar (Becton Dickinson and Company)), and incubated in atmosphere supplemented with 5% CO_2_ at 37 °C in a humidified incubator. The antibiotics rifampicin (100 µg/mL), nalidixic acid (50 µg/mL), and spectinomycin (50 µg/mL) were added when appropriate. In some experiments the bacteria were cultured in RPMI (Sigma, St. Louis, Missouri, USA, Catalog #: R0883), or in a keratinocyte medium (Green’s medium) [48] consisted of 63% Dulbecco’s modified Eagle’s medium (DMEM, Life Technologies, Paisley, UK) supplemented with 0.14% NAHCO_3_ and 13mM Hepes, 25% Ham-F-12-medium (Life Technologies), 10% Fetal bovine serum (Life Technologies), 4 mM L-glutamine (Life Technologies), 5 µg/mL Insulin (Sigma), 0.4 µg/mL Hydrocortisone (Sigma), 5 ng/mL Epidermal growth factor, EGF (Sigma), 0.1 nM Cholera toxin (Sigma), 1.8 µg/mL Adenine (Sigma) and 100 µg/mL freshly added Ascorbic acid (Sigma). 

### 4.2. Transcriptomic Analysis via RNA-Seq

Transcriptomic analysis of log-phase bacteria was performed in 4 experimental conditions, each with three biological replicates. These included the growth of planktonic strain D7SS in mTSB, the growth of biofilm-forming strain D7S-1 in mTSB, RPMI and Green’s medium. The starter bacterial cultures were prepared by transferring 10–15 colonies of bacteria from agar into 5 ml of mTSB and incubated overnight in atmosphere supplemented with 5% CO_2_ at 37 °C in a humidified incubator. The colony forming unit/ml was estimated based on optical density (OD_600_ = 1 is equivalent to 10^9^/mL). 

An aliquot (0.2–0.4 mL) of the bacterial culture containing 10^8^ CFU was transferred into each well of a polystyrene 6-well tissue culture plate (Multiwell^TM^, Becton Dickinson, New Jersey, USA), and 3 mL of fresh mTSB was added to each well. The plate was then incubated for 20 h. For biofilm-forming D7S-1, the culture supernatant was removed and the biofilm attached to the bottom of the well was gently rinsed with warm fresh medium once, and then 2 mL of fresh mTSB, RPMI or keratinocyte medium was added, and incubated for 6 h. Afterward, the supernatant was removed, and 0.7 mL of RNA*later*^®^ (ThermoFisher Scientific, Waltham, Massachusetts, USA) was added to each well. The bacterial cells were then collected with the aid of a cell scraper (Greiner Bio-One, Monroe, North Carolina, USA), pelleted by centrifugation at 10,000 rpm for 2 min, kept at 4 °C for one hour, and then stored at −80 °C until used. 

For the non-biofilm forming planktonic D7SS, after the same 20-hour incubation, 2 mL of the culture was removed (leaving 1 mL of the overnight culture), replaced with 2 mL of pre-warmed fresh mTSB, and incubated for 6 h. At the end of the 6-hour incubation, OD_600_ was measured again to assure that the bacteria were still in the log phase. Next, 1 mL of RNA*later*^®^ was added into each well, and bacterial cells were harvested as above. After the supernatant was discarded, 0.7 mL of RNAlater^®^ was added to the cells, incubated at 4 °C for 1 h, and then stored at −80 °C until use. 

Total RNA was extracted using the Ribo-Pure Bacterial RNA isolation kit following the manufacturer’s instructions (Life Technology, Grand Island, NY, USA). Briefly, 1.0 × 10^9^ cells were lysed using zirconia beads, and the lysate was mixed with chloroform. The RNA was extracted in the top aqueous phase, cleaned, and treated with DNase to prepare for RNA sequencing. The purified mRNA was fragmented using divalent cations at elevated temperature. Cleaved RNA fragments were copied into first-strand cDNA using reverse transcriptase and random primers, followed by second-strand cDNA synthesis using DNA polymerase I and RNase H. cDNA products were purified and enriched by PCR to create a final cDNA library using the TruSeq Stranded Total RNA sample preparation kit (Illumina, San Diego, CA, USA). After sequencing, the reads for each sample were mapped to the corresponding genomes for each strain using the Geneious software (Biomatters LTD, Auckland, New Zealand). After mapping, the average coverage (number of sequences/nucleotide) was calculated for each predicted gene. Coverage was normalized by averaging across all genes for each sample and scaled up by multiplying by a factor of 1000. These RNA-Seq data are available via BioProject accession number PRJNA575215.

The replication and the viability of *A. actinomycetemcomitans* biofilms in tested media were evaluated. The replication of *A. actinomycetemcomitans* was examined by the measuring the optical density of the cell cultures at 600 nm. Briefly, the bacteria were collected from agar plates, resuspended in the media and adjusted to approximately 10^8^ CFU/mL. The optical density of the cultures was recorded for 48 h. The viability of *A. actinomycetemcomitans* in the tested media was determined by enumerating CFU of the cultures. Briefly, biofilms were prepared in tissue culture wells as described above and incubated in each of the tested media. At specific time points, the cells were collected, serially diluted in the media and plated on mTSB agar. The plates were incubated in atmosphere supplemented with 5% CO_2_ at 37 °C in a humidified incubator for 3–4 days to enumerate CFU. All experiments were performed in biological triplicates.

### 4.3. Metabolic Network 

To determine pathways affected by the differentially expressed genes, we used the KEGG Mapper Search and Color Pathway tool as previously described [24,49] using the locus tag of each differentially expressed genes obtained from each media. A comparison of the pathways affected were then attempted. 

### 4.4. Quantitative Real-Time PCR (qRT-PCR)

The relative gene expression levels by RNA-Seq were confirmed by qRT-PCR using BioRad iCycler iQ® Real-Time PCR Detection System as described previously [16] for the following genes: *cdtA* (D7S_02294)*, cdtB* (D7S_02295), *ltxA* (D7S_00604)*,* and *metF* (D7S_00244). A constitutively expressed house-keeping gene *clpX* (D7S_01693) was used as a reference to compare the expression levels [50]. For each sample, 1 µg of RNA in a 20 µL reaction mixture was reverse transcribed into first strand cDNA using SuperScript VILO kit (ThermoFisher Scientific). Reactions without reverse transcriptase or RNA template were included as controls. The first strand cDNA synthesis was performed at 25 °C for 10 min, 42 °C for 60 min, and 85 °C for 5 min. The 20 µL volume containing the cDNA was then diluted to 200 µL using sterile water. For qRT-PCR, a volume of 2 µL of the diluted cDNA from each sample was used following the protocol described by the manufacturer. Briefly, the reaction mixture included 2.5 µL of each primer (3 µM), 12.5 µL of 2X iQ SYBR Green Supermix (BioRad, Hercules, California, USA), 2 µL of cDNA, and water to 25 µL. The thermocycling profile consisted of four cycles as follows: Cycle 1: (1X) Step 1: 95 °C for 3 min. Cycle 2: (40X) Step 1: 95 °C for 10 s. Step 2: 55 °C for 30 s. Cycle 3: (1X) Step 1: 95 °C for 1 min. Cycle 4: (1X) Step 1: 55 °C for 1 min. For the melting curves, the final DNA products were denatured at 95 °C for 1 min. and then incubated at 5 °C below the annealing temperature for 1 min. before the temperature was increased to 95 °C at a ramp rate of 0.5 °C/10 s. For each sample, both target gene and reference gene were done in triplicate. Additional controls included samples without cDNA for each target gene. Data analysis was performed based on the protocol provided by BioRad. The transcript levels of the genes of interest were normalized to the transcript level of the house-keeping gene, *clpX*.

### 4.5. Statistical Analysis

Student’s *t*-test was performed to compare the levels of transcripts in different gene categories (core, accessory and island) at *p <* 0.01. Differentially expressed genes were identified by Student’s *t*-test at *p* < 0.05 and 1.5 or greater fold changes. The correlation between the expression levels by RNA-seq and qRT-PCR was determined by linear regression. Statistical analysis to determine the significance of the pathways affected by the differentially expressed genes was performed using the computing environment R [51].

## Figures and Tables

**Table 1:** Differentially expressed A. actinomycetemcomitans genes in different growth conditions.

		Up 1.5-Fold **			Down 1.5-Fold **	
Growth Condition *	Core	Non-Island Accessory Genes	Island Genes	Core	Non-Island Accessory Genes	Island Genes
Planktonic in mTSB	66	4	9	143	10	10
Biofilm in Keratinocyte Medium	454	53	72	145	13	13
Biofilm in RPMI	515	115	158	352	22	17

* As compared to biofilm in modified Trypticase Soy Broth (mTSB) control. ** Statistically significant at p < 0.05 by Student’s t-test.

## Supplementary Materials

The following are available online at https://www.mdpi.com/2076-0817/8/4/282/s1, Figure S1: Correlations of genes expression levels obtained by qRT-PCR and RNA-Seq, Figure S2: Changes in optical density and CFU comparisons between *A. actinomycetemcomitans* cultured in rich mTSB, RPMI, and keratinocyte medium, Table S1: *A. actinomycetemcomitans* Genomic Islands, Table S2: Student’s *t*-test of Gene Expression Levels between Gene Categories in All Tested Conditions, Table S3: Mean and Median Values of Gene Expression Levels of Core and Island Genes in Different Growth Conditions, Table S4: Upregulated and Downregulated *A. actinomycetemcomitans* Gene Expressions in Different Growth Conditions.
