# Supplementary material for: Transcriptomic Analysis of Aggregatibacter actinomycetemcomitans Core and Accessory Genes in Different Growth Conditions
Source: Pathogens. 2019 Dec 3;8(4):282. doi: 10.3390/pathogens8040282 (PMC6963384; doi:10.3390/pathogens8040282)
Supplement: Supplementary file 1 [file pathogens-08-00282-s001.zip › New folder/Supplementary Figure S1.FINAL.Rev.docx]

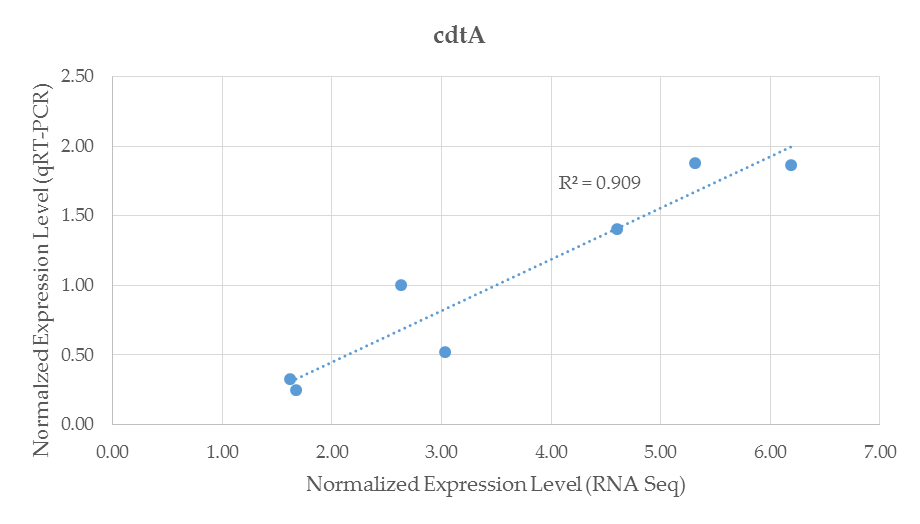


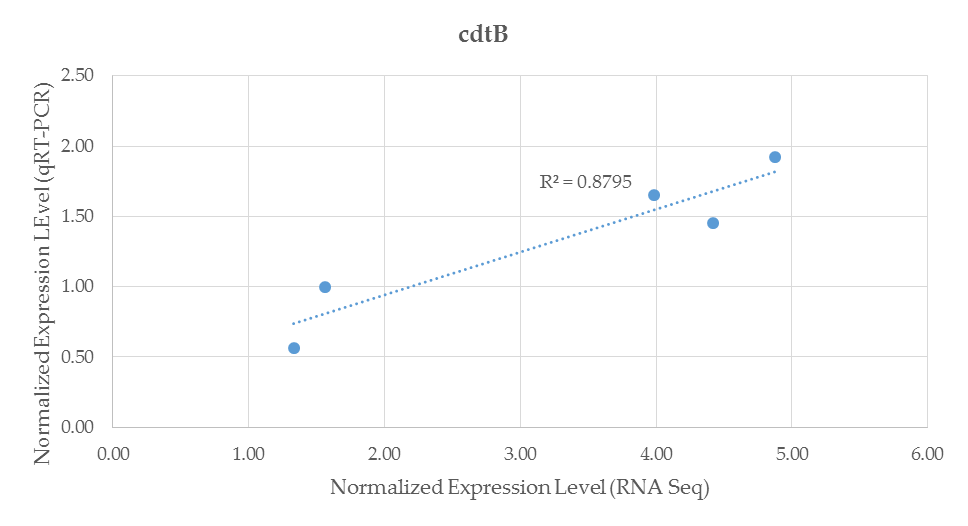


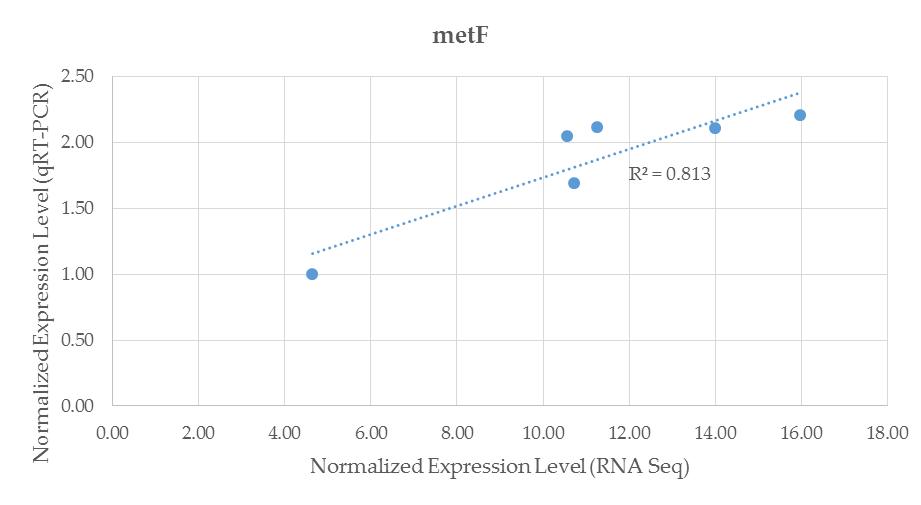


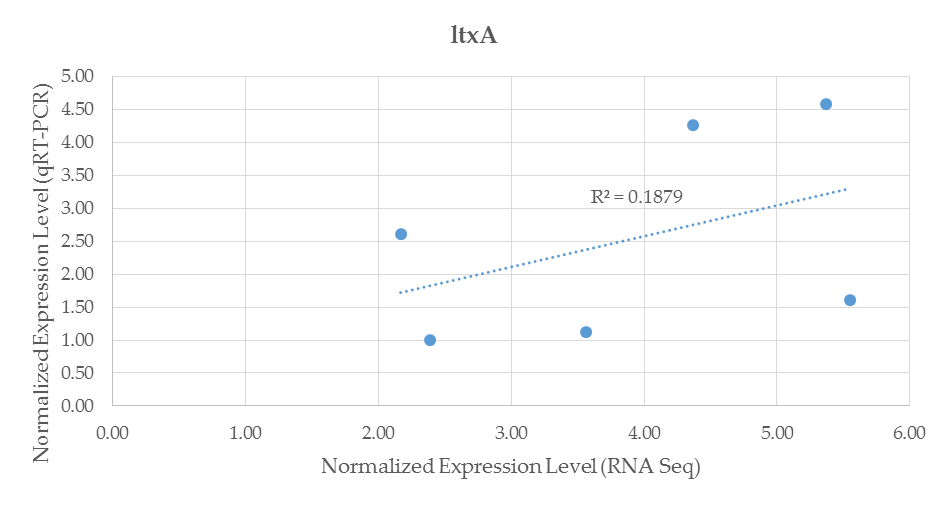


**Supplementary Figure S1**. Correlations of genes expression levels obtained by qRT-PCR and RNA Seq. qRT-PCR was performed on selected genes and excellent correlations between qRT-PCR and RNA Seq results (R=0.81 – 0.91) were observed. Poor correlation was discovered for *ltxA* gene, and this had been observed previously.
