# Supplementary material for: Transcriptomic Analysis of Aggregatibacter actinomycetemcomitans Core and Accessory Genes in Different Growth Conditions
Source: Pathogens. 2019 Dec 3;8(4):282. doi: 10.3390/pathogens8040282 (PMC6963384; doi:10.3390/pathogens8040282)
Supplement: Supplementary file 1 [file pathogens-08-00282-s001.zip › New folder/Supplementary Figure S2.FINAL.Rev.2.docx]

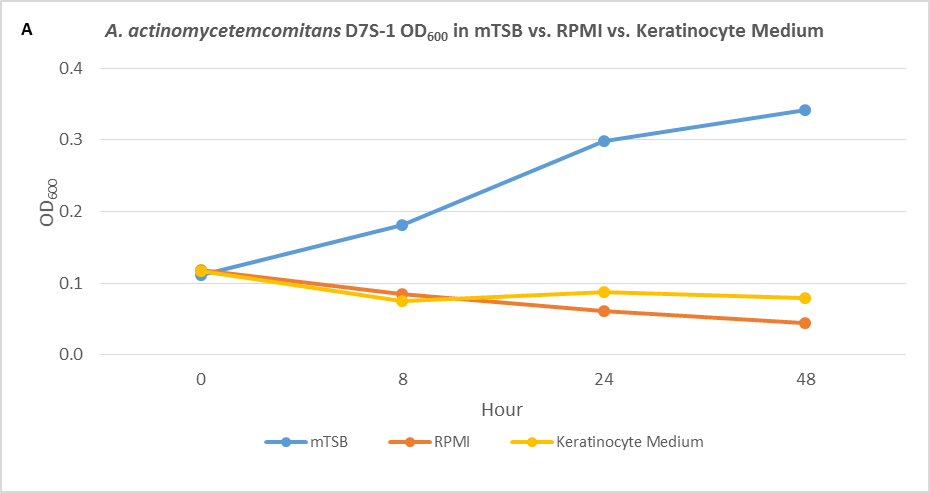


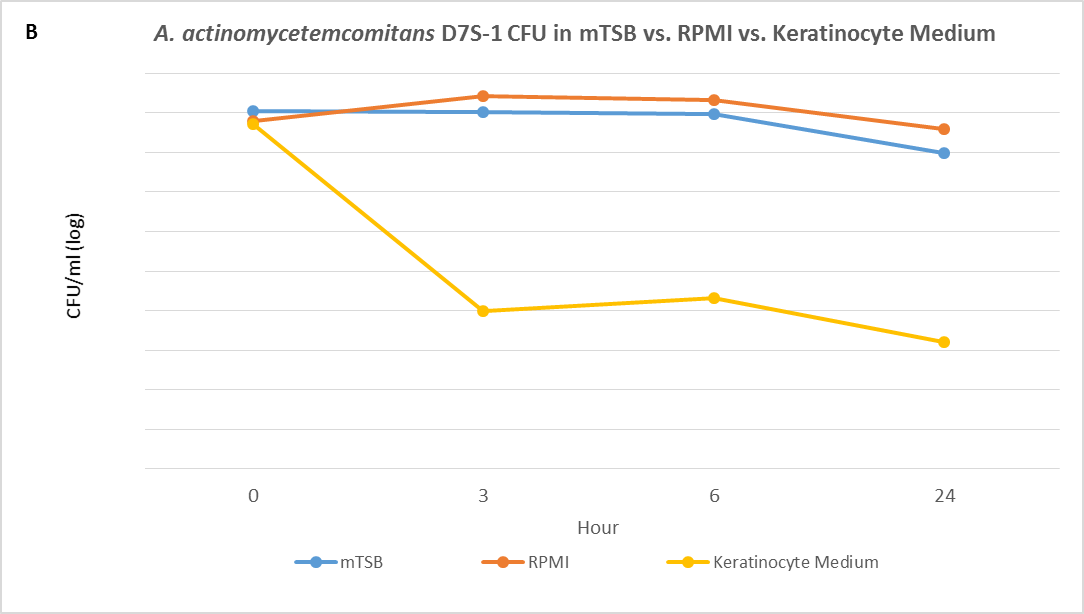


**Supplementary Figure S2**. **A**. Changes in optical density when *A. actinomycetemcomitans* D7S-1 were cultured in either enriched mTSB or nutrient limited RPMI or keratinocyte medium. *A. actinomycetemcomitans* D7S-1 grew in mTSB medium, but did show any evidence of growth in both RPMI and keratinocyte medium. **B.** CFU comparisons between *A. actinomycetemcomitans* D7S-1 cultured in enriched mTSB, RPMI, and keratinocyte medium. Serial dilutions of bacterial cultures were made at different time points and CFUs were calculated. *A. actinomycetemcomitans* D7S-1 grew in the enriched mTSB (as expected), maintained its viability in RPMI without growth, but showed a reduced viability in keratinocyte medium.
