# Supplementary material for: Transcriptomic Analysis of Aggregatibacter actinomycetemcomitans Core and Accessory Genes in Different Growth Conditions
Source: Pathogens. 2019 Dec 3;8(4):282. doi: 10.3390/pathogens8040282 (PMC6963384; doi:10.3390/pathogens8040282)
Supplement: Supplementary file 1 [file pathogens-08-00282-s001.zip › New folder/Supplementary Table S1.FINAL.Rev.docx]

**Supplementary Table S1 – Genomic Islands of *Aggregatibacter actinomycetemcomitans* D7S-1.**

Each genomic island information starts with a line showing the island number (e.g. Island 1) followed by a line describing its location in the genome. For example, D7S-1 CP003496 (181635 - 186853) means that this genomic island begins at position 181635 and ends at position 186853 of D7S-1 genome with accession number CP003496. Subsequent lines display the gene content found in each island. The columns in the gene content lines are gene ID, length of protein product, product description, and %GC value. The symbol *, **, or *** after the %GC value denotes that the %GC value is 1-2, 2-3 or more than 3 standard deviations from the mean %GC value of the genome. Genomic islands with gene content exhibiting highly deviated base composition values (e.g. %GC value) may have been acquired by horizontal gene transfer.

Island 1

D7S-1 CP003496 (181635 - 186853)

D7S_00175 788 aa TonB-dependent receptor 38%*

D7S_00176 924 aa peptidase M16 39%*

Island 2

D7S-1 CP003496 (192314 - 202600)

D7S_00181 97 aa CRISPR-associated protein Cas2 46%

D7S_00182 337 aa CRISPR-associated protein Cas1 50%*

D7S_00185 215 aa CRISPR-associated protein Cas4 58%**

D7S_00186 297 aa CRISPR-associated protein Csd2 52%*

D7S_00187 677 aa CRISPR-associated protein Csd1 44%

D7S_00188 259 aa CRISPR-associated protein 48%*

D7S_00190 821 aa CRISPR-associated protein 49%*

Island 3

D7S-1 CP003496 (288191 - 295534)

D7S_00288 276 aa 5-deoxyglucuronate isomerase 45%

D7S_00291 384 aa hypothetical protein 45%

D7S_02605 367 aa phospholipase 45%

D7S_02610 379 aa phospholipase 42%*

D7S_00294 740 aa type IV secretion protein Rhs 48%*

Island 4

D7S-1 CP003496 (414680 - 424326)

D7S_00411 638 aa type IV secretion protein Rhs 49%*

D7S_02665 142 aa hypothetical protein 41%*

D7S_02670 238 aa type IV secretion protein Rhs 44%

D7S_00414 159 aa calcium-binding protein 33%**

D7S_00415 236 aa hypothetical protein 24%***

D7S_00416 314 aa glycoside hydrolase family 19 39%*

D7S_02675 85 aa hypothetical protein 30%***

D7S_00417 210 aa hypothetical protein 27%***

D7S_02680 83 aa hypothetical protein 25%***

D7S_00420 195 aa hypothetical protein 25%***

D7S_02685 91 aa hypothetical protein 33%**

D7S_00423 59 aa restriction endonuclease subunit R 37%*

Island 5

D7S-1 CP003496 (508580 - 515504)

D7S_00518 211 aa transcriptional regulator 38%*

D7S_00519 128 aa DNA repair protein 34%**

D7S_00520 357 aa chemotaxis protein 38%*

D7S_00521 551 aa ATPase 32%**

D7S_00522 448 aa Fic family protein 36%**

D7S_00525 179 aa cell division protein FtsZ 41%*

Island 6

D7S-1 CP003496 (534905 - 545339)

D7S_00541 502 aa methylmalonate-semialdehyde dehydrogenase 46%

D7S_00543 448 aa CRISPR-associated protein 37%*

D7S_00544 319 aa CRISPR-associated protein Csy2 41%*

D7S_00545 336 aa CRISPR-associated protein Cas5 41%*

D7S_00546 187 aa CRISPR-associated protein Csy4 43%

D7S_00547 1095 aa helicase 42%*

D7S_00548 321 aa CRISPR-associated protein Cas1 43%

Island 7

D7S-1 CP003496 (578920 - 593439)

D7S_00587 217 aa hypothetical protein 29%***

D7S_00588 172 aa mannosyl-glycoprotein endo-beta-N-acetylglucosamidase 34%**

D7S_00591 62 aa type VI secretion protein VgrG 46%

D7S_00593 279 aa Fe-S cluster assembly protein HesB 42%*

D7S_00594 636 aa 5-dehydro-2-deoxygluconokinase 48%*

D7S_00596 648 aa 3D-(3,5/4)-trihydroxycyclohexane-1,2-dione hydrolase 50%*

D7S_00597 298 aa inosose dehydratase 45%

D7S_00598 336 aa oxidoreductase 51%*

D7S_00599 170 aa mannitol repressor protein 42%*

D7S_00600 382 aa mannitol-1-phosphate 5-dehydrogenase 49%*

D7S_00601 626 aa PTS system mannitol-specific transporter subunit IICBA 48%*

Island 8

D7S-1 CP003496 (669167 - 676230)

D7S_00690 68 aa hypothetical protein 40%*

D7S_02805 194 aa hypothetical protein 39%*

D7S_02810 193 aa hypothetical protein 40%*

D7S_00695 988 aa hypothetical protein 43%

D7S_00696 470 aa signal peptide protein 45%

D7S_00697 41 aa transposase 47%

D7S_00698 66 aa integrase 41%*

Island 9

D7S-1 CP003496 (736023 - 741308)

D7S_02855 75 aa hypothetical protein 43%

D7S_00756 831 aa hypothetical protein 40%*

D7S_02860 113 aa hypothetical protein 35%**

D7S_02865 354 aa hypothetical protein 43%

Island 10

D7S-1 CP003496 (869666 - 876458)

D7S_00913 783 aa hypothetical protein 35%**

D7S_00914 335 aa hypothetical protein 34%**

D7S_00915 75 aa hypothetical protein 42%*

D7S_00916 193 aa molybdenum cofactor biosynthesis protein MogA 53%*

D7S_00917 309 aa phosphatidate cytidylyltransferase 48%*

D7S_00918 210 aa acyltransferase 47%

D7S_00919 202 aa CDP-alcohol phosphatidyltransferase 46%

Island 11

D7S-1 CP003496 (953929 - 958945)

D7S_00994 369 aa alpha/beta hydrolase 49%*

D7S_00995 551 aa carboxylesterase 45%

D7S_00996 326 aa aldehyde oxidase 50%*

D7S_00997 281 aa 2,5-diketo-D-gluconic acid reductase 47%

Island 12

D7S-1 CP003496 (1049542 - 1055050)

D7S_03020 175 aa hypothetical protein 45%

D7S_03025 100 aa membrane protein 50%*

D7S_01107 147 aa DNA-binding protein 47%

D7S_01108 371 aa acriflavin resistance protein AcrA 48%*

D7S_01109 62 aa hypothetical protein 46%

D7S_01110 723 aa prepilin peptidase 30%***

Island 13

D7S-1 CP003496 (1097673 - 1111699)

D7S_03065 740 aa type IV secretion protein Rhs 48%*

D7S_01167 384 aa hypothetical protein 45%

D7S_01169 161 aa dihydrofolate reductase 51%*

D7S_01170 154 aa membrane protein 51%*

D7S_01171 261 aa hypothetical protein 52%*

D7S_03075 59 aa glutamate 5-kinase 49%*

D7S_03080 221 aa glutamate 5-kinase 51%*

D7S_01174 210 aa peptidylprolyl isomerase 50%*

D7S_01175 289 aa phosphatidylserine decarboxylase 48%*

D7S_01176 429 aa adenosylmethionine-8-amino-7-oxononanoate aminotransferase 44%

D7S_01177 391 aa 8-amino-7-oxononanoate synthase 40%*

D7S_01178 216 aa hypothetical protein 42%*

Island 14

D7S-1 CP003496 (1173232 - 1194196)

D7S_03100 99 aa hypothetical protein 36%**

D7S_03105 111 aa hypothetical protein 43%

D7S_03110 696 aa hypothetical protein 46%

D7S_01255 52 aa membrane protein 43%

D7S_01257 601 aa type IV secretion protein Rhs 47%

D7S_01258 470 aa signal peptide protein 45%

D7S_01259 988 aa hypothetical protein 43%

D7S_01260 193 aa hypothetical protein 40%*

D7S_01265 108 aa type IV secretion protein Rhs 46%

D7S_01266 319 aa signal peptide protein 40%*

D7S_01267 934 aa hypothetical protein 40%*

D7S_01268 288 aa hypothetical protein 41%*

D7S_01270 284 aa hypothetical protein 45%

D7S_01272 64 aa ABC transporter permease 31%**

D7S_01273 145 aa hypothetical protein 28%***

D7S_01274 177 aa hypothetical protein 32%**

D7S_03125 197 aa hypothetical protein 30%***

Island 15

D7S-1 CP003496 (1205216 - 1219692)

D7S_01289 463 aa major facilitator transporter 52%*

D7S_01290 430 aa mannose-1-phosphate guanyltransferase 37%*

D7S_03130 263 aa ABC transporter 25%***

D7S_03135 250 aa teichoic acid ABC transporter ATP-binding protein 33%**

D7S_01294 977 aa glycosyl transferase 30%***

D7S_01295 295 aa epimerase 31%**

D7S_01296 397 aa acetyltransferase 27%***

D7S_01297 344 aa GDP-D-mannose dehydratase 35%**

D7S_01298 390 aa glycosyl transferase 29%***

D7S_01299 363 aa glycosyl transferase 38%*

D7S_01300 472 aa UDP-phosphate galactose phosphotransferase 36%**

Island 16

D7S-1 CP003496 (1245945 - 1256964)

D7S_01329 375 aa cell division protein Fic 45%

D7S_01330 308 aa ADP-L-glycero-D-manno-heptose-6-epimerase 52%*

D7S_01331 258 aa hypothetical protein 36%**

D7S_01332 604 aa metallophosphatase 38%*

D7S_01333 391 aa hypothetical protein 35%**

D7S_01334 763 aa hypothetical protein 39%*

D7S_01336 464 aa signal peptide protein 46%

Island 17

D7S-1 CP003496 (1311321 - 1331186)

D7S_01398 647 aa hypothetical protein 47%

D7S_03180 83 aa hypothetical protein 31%**

D7S_01399 376 aa hypothetical protein 39%*

D7S_03185 62 aa integrase 41%*

D7S_01401 733 aa phospholipase 44%

D7S_01402 384 aa hypothetical protein 45%

D7S_01403 390 aa hypothetical protein 45%

D7S_01406 169 aa hypothetical protein 43%

D7S_01407 68 aa hypothetical protein 40%*

D7S_01408 123 aa hypothetical protein 40%*

D7S_01409 232 aa hypothetical protein 43%

D7S_01412 141 aa hypothetical protein 41%*

D7S_01413 254 aa antirepressor 44%

D7S_01414 91 aa hypothetical protein 41%*

D7S_01415 127 aa terminase 50%*

D7S_01416 103 aa hypothetical protein 42%*

D7S_01417 150 aa hypothetical protein 41%*

D7S_01418 86 aa transcriptional regulator 44%

D7S_01419 69 aa regulatory protein 42%*

D7S_01420 258 aa hypothetical protein 38%*

D7S_01421 223 aa hypothetical protein 31%**

D7S_01422 414 aa integrase 43%

D7S_01423 75 aa hypothetical protein 42%*

Island 18

D7S-1 CP003496 (1603785 - 1611311)

D7S_03385 129 aa transposase 52%*

D7S_03390 93 aa transposase 47%

D7S_01716 706 aa hypothetical protein 45%

D7S_01720 228 aa peptidase A24 48%*

D7S_01721 407 aa type II secretion system protein F 48%*

Island 19

D7S-1 CP003496 (1630899 - 1636995)

D7S_01741 238 aa hypothetical protein 53%*

D7S_03405 396 aa hypothetical protein 44%

D7S_03410 529 aa hypothetical protein 45%

D7S_01745 392 aa signal peptide protein 42%*

Island 20

D7S-1 CP003496 (1775233 - 1781380)

D7S_01898 57 aa transposase 43%

D7S_01901 425 aa hypothetical protein 42%*

D7S_03490 251 aa hypothetical protein 47%

D7S_03495 353 aa hypothetical protein 45%

D7S_01904 327 aa hypothetical protein 41%*

Island 21

D7S-1 CP003496 (1796374 - 1806441)

D7S_01920 568 aa 2-succinyl-5-enolpyruvyl-6-hydroxy-3- cyclohexene-1-carboxylate synthase 51%*

D7S_01921 249 aa 2-succinyl-6-hydroxy-2, 4-cyclohexadiene-1-carboxylate synthase 47%

D7S_01922 89 aa 30S ribosomal protein S15 41%*

D7S_01923 384 aa S-adenosylmethionine synthetase 50%*

D7S_01926 57 aa transposase 43%

D7S_01928 311 aa hypothetical protein 40%*

D7S_03515 838 aa hypothetical protein 46%

D7S_03520 111 aa hypothetical protein 43%

D7S_03525 99 aa hypothetical protein 36%**

Island 22

D7S-1 CP003496 (1987841 - 2005285)

D7S_03595 144 aa CRISPR-associated protein Cas3 57%**

D7S_03600 567 aa CRISPR-associated protein Cas3 58%**

D7S_02118 958 aa DEAD/DEAH box helicase 51%*

D7S_03605 193 aa hypothetical protein 34%**

D7S_02120 313 aa hypothetical protein 32%**

D7S_02121 578 aa DNA methylase N-4 46%

D7S_02123 344 aa toxin Fic 45%

D7S_02124 905 aa type III restriction enzyme, res subunit 49%*

D7S_02125 224 aa CRISPR-associated protein 56%**

D7S_03610 465 aa CRISPR-associated protein Csd1 56%**

D7S_03615 127 aa hypothetical protein 54%**

D7S_02128 288 aa CRISPR-associated protein Csd2 53%*

D7S_02129 216 aa CRISPR-associated protein Cas4 58%**

D7S_02130 234 aa hypothetical protein 36%**

Island 23

D7S-1 CP003496 (2044444 - 2050326)

D7S_02163 430 aa hypothetical protein 45%

D7S_03635 114 aa hypothetical protein 26%***

D7S_03640 107 aa hypothetical protein 27%***

D7S_02164 561 aa formate dehydrogenase 47%

D7S_02165 141 aa spermidine/putrescine ABC transporter substrate-binding protein 45%

D7S_02166 397 aa cell filamentation protein 36%**

Island 24

D7S-1 CP003496 (2154017 - 2178164)

D7S_02275 495 aa N-acetylmuramoyl-L-alanine amidase 44%

D7S_02276 164 aa ATP-binding protein 41%*

D7S_02277 597 aa membrane protein 30%***

D7S_02280 306 aa glycosyl transferase family 2 32%**

D7S_02281 502 aa membrane protein 33%**

D7S_02282 205 aa membrane protein 36%**

D7S_02283 541 aa Dca 30%***

D7S_02284 273 aa integrase 32%**

D7S_02285 636 aa relaxase 36%**

D7S_02286 59 aa hypothetical protein 34%**

D7S_02287 92 aa hypothetical protein 33%**

D7S_02288 59 aa hypothetical protein 36%**

D7S_02292 114 aa MazF family transcriptional regulator 41%*

D7S_02293 88 aa virulence protein 39%*

D7S_02294 222 aa cytolethal distending toxin protein A (CdtA) 39%*

D7S_02295 283 aa cytolethal distending toxin subunit CdtB 41%*

D7S_02296 186 aa cytolethal distending toxin protein C (CdtC) 38%*

D7S_02297 63 aa antirestriction protein 42%*

D7S_02298 253 aa hypothetical protein 33%**

D7S_02302 107 aa hypothetical protein 44%

D7S_02303 59 aa hypothetical protein 46%

D7S_02304 135 aa membrane protein 41%*

D7S_02305 123 aa membrane protein 41%*

D7S_02306 210 aa hypothetical protein 45%

D7S_02307 286 aa conjugal transfer protein 46%

D7S_02308 462 aa hypothetical protein 45%

D7S_02309 135 aa conjugal transfer protein 48%*

Island 25

D7S-1 CP003496 (2229685 - 2243325)

D7S_02366 95 aa peptidase 41%*

D7S_02367 98 aa LacI family transcriptional regulator 41%*

D7S_02369 66 aa integrase 41%*

D7S_02370 41 aa transposase 47%

D7S_02371 464 aa signal peptide protein 46%

D7S_02374 763 aa hypothetical protein 39%*

D7S_02375 391 aa hypothetical protein 35%**

D7S_02376 394 aa permease 48%*

D7S_02377 228 aa ABC transporter ATP-binding protein 48%*

D7S_02378 416 aa outer membrane-specific lipoprotein transporter subunit LolE 47%

D7S_02379 359 aa phospho-2-dehydro-3-deoxyheptonate aldolase 45%

D7S_02381 468 aa DNA helicase 46%

Island 26

D7S-1 CP003496 (2296615 - 2307183)

D7S_03775 165 aa hypothetical protein 39%*

D7S_02436 333 aa iron ABC transporter substrate-binding protein 49%*

D7S_02437 337 aa iron ABC transporter permease 49%*

D7S_02438 249 aa iron ABC transporter ATP-binding protein 47%

D7S_03780 100 aa hypothetical protein 48%*

D7S_03785 502 aa TonB-dependent receptor 43%

D7S_02441 69 aa molybdenum-pterin-binding protein 44%

D7S_02442 344 aa peptide ABC transporter substrate-binding protein 45%

D7S_02443 262 aa cobalamin ABC transporter ATPase 45%

D7S_02444 328 aa iron ABC transporter permease 51%*

D7S_03790 231 aa hypothetical protein 39%*

D7S_02447 90 aa tail fiber assembly protein 40%*
