# Supplementary material for: Transcriptomic Analysis of Aggregatibacter actinomycetemcomitans Core and Accessory Genes in Different Growth Conditions
Source: Pathogens. 2019 Dec 3;8(4):282. doi: 10.3390/pathogens8040282 (PMC6963384; doi:10.3390/pathogens8040282)
Supplement: Supplementary file 1 [file pathogens-08-00282-s001.zip › New folder/Supplementary Table S3.FINAL.Rev.docx]

**Supplementary Table S3.** Mean and Median Values of Gene Expression Levels^a^ of Core and Island Genes in Different Growth Conditions

|  | **Core Genes** | | **Island Genes** | |
| --- | --- | --- | --- | --- |
| **Growth Condition** | **Mean (SD^b^)** | **Median** | **Mean (SD^b^)** | **Median** |
| Biofilm in mTSB (control) | 7.68 (1.99) | 7.61 | 5.83 (1.93) | 5.86 |
| Biofilm in Keratinocyte medium | 7.95 (1.96) | 7.89 | 6.27 (1.85) | 6.40 |
| Biofilm in RPMI | 7.87 (1.77) | 7.79 | 7.01 (1.94) | 7.12 |
| Planktonic in mTSB | 7.63 (1.98) | 7.53 | 5.93 (2.01) | 6.10 |

^a^Log_2_ (transcripts) ^b^Standard Deviation
